# State of the Art Therapie mit Impella® in der Herzchirurgie in Österreich

**DOI:** 10.1007/s00508-024-02407-4

**Published:** 2024-09-09

**Authors:** Dominik Wiedemann, Julia Dumfarth, Andreas F. Zierer, Daniel Zimpfer

**Affiliations:** 1 Klinische Abteilung für Herzchirurgie, Universitätsklinikum St. Pölten, Karl Landsteiner Privatuniversität für Gesundheitswissenschaften, St. Pölten, Österreich; 2 Universitätsklinik für Herzchirurgie, Medizinische Universität Innsbruck, Innsbruck, Österreich; 3 Abteilung für Herz-, Gefäß- und Thoraxchirurgie, Johannes Kepler Universität Linz, Linz, Österreich; 4 Universitätsklinik für Herzchirurgie, Medizinische Universität Wien, Wien, Österreich

**Keywords:** Impella, Mechanische Herz-Kreislauf-Unterstützung, Herzinsuffizienz, Mittelfristige Herzunterstützung, Herzerholung, Impella, Mechanical Circulatory suppor, Heart failure, Medium-term cardiac support, Heart recovery

## Abstract

Seit 2022 wird in Österreich das mechanische Linksherzunterstützungssystem Impella 5.5® zur Versorgung von Patienten mit kardiogenem Schock, bei fortgeschrittener Herzinsuffizienz, Postkardiotomie und Low-Cardiac-Output-Syndrom eingesetzt. Die chirurgische Einbringung der Impella 5.5 über die Arteria subclavia oder alternativ über die Aorta ascendens ist inzwischen in Österreich ein etabliertes Verfahren zur mittelfristigen Therapie von Patienten im kardiogenen Schock und für Bridging-Szenarien, wie z. B. „bridge to recovery“, „bridge to linksventrikulärem Assist Device“ (LVAD), „bridge to decision“ und „bridge to heart transplant“ (HTx). Allen linksventrikulären Impella-Herzpumpen ist gemein, dass sie den linken Ventrikel entlasten, wobei die Impella 5.5 ein volles Herzzeitvolumen von 5,5 l/min erreicht. Aufgrund der stabilen Lage mittels der transaxillären oder transaortalen Insertionstechnik sind eine rasche Extubation und Mobilisierung des Patienten auf der Intensivstation (Intensive Care Unit, ICU) möglich. Dies führt in weiterer Folge zu einer deutlichen Verkürzung des ICU-Aufenthalts. Auch eine Kombination von Impella 5.5 mit einer venoarteriellen extrakorporalen Membranoxygenierung (VA-ECMO) hat sich in verschiedenen Fällen als wirksam erwiesen. Eine Reihe an nicht randomisierten Studien weist die Wirksamkeit und Sicherheit der Impella 5.5 in der Praxis nach, die in mehreren internationalen Guidelines Eingang fanden. Die Vorteile der Impella 5.5 in der Praxis sind die einfache Handhabung mit hoher Lagestabilität und niedrige Komplikationsraten. Diese Veröffentlichung beschreibt den Stellenwert der chirurgischen Impella-Therapie in Österreich aus der Sicht der klinischen Experten aus Österreich.

## Abstract (en)

Since 2022, the mechanical left ventricular support system Impella 5.5® has been used in Austria for patients with cardiogenic shock, advanced heart failure, post-cardiotomy and low output syndrome. The surgical insertion of the Impella 5.5 via the subclavian artery or alternatively via the ascending aorta has become an established procedure for medium-term treatment in patients with cardiogenic shock and bridging scenarios, such as bridge to recovery, bridge to left ventricular assist device (LVAD), bridge to decision, and bridge to heart transplant (HTx) in Austria. All Impella left ventricular heart pumps share the common feature of unloading the left ventricle, with the Impella 5.5 achieving a full cardiac output of 5.5 l/min. The stable positioning via transaxillary or transaortic insertion enables rapid extubation and mobilization of patients in the intensive care unit (ICU), leading to a significantly shorter ICU stay. The combined support of Impella 5.5 with venoarterial extracorporeal membrane oxygenation (VA-ECMO) has also proven effective in certain scenarios. Several nonrandomized studies demonstrated the effectiveness and safety of the Impella 5.5 in practice, which have been included in multiple international guidelines. The advantages of the Impella 5.5 in practice include the easy handling with high positional stability, and low complications rates. This article describes the significance of surgical Impella treatment in Austria from the perspective of Austrian clinical experts.

## Einleitung

**Abb. 1:**
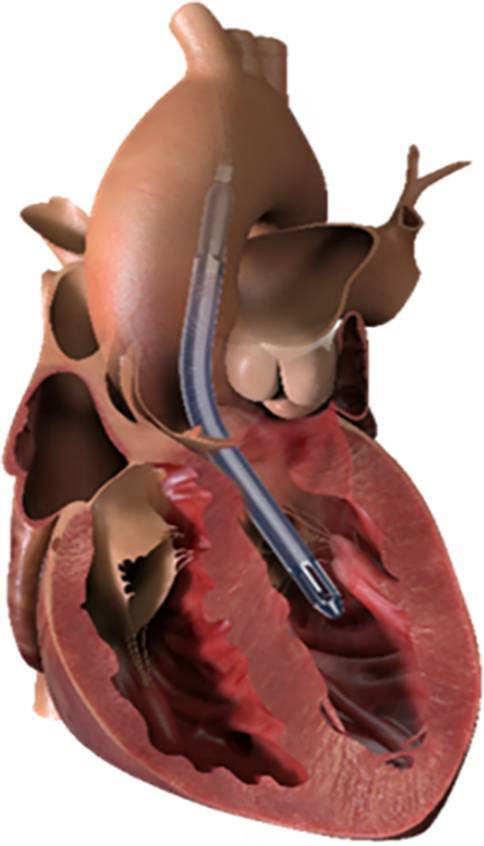
Die Impella 5.5. Die Impella 5.5 (Abiomed, Aachen, Deutschland) erreicht ein HZV von bis zu 5,5 l/min und entlastet den linken Ventrikel. (Quelle der Abbildung: https://www.abiomed.de/ueber-uns/presse-und-news/media-kit)

Temporäre mechanisch zirkulatorische Unterstützungssysteme (tMCS) können ein adäquates Herzzeitvolumen (HZV) und somit die Organperfusion wiederherstellen [1–3]. Derzeit in Österreich eingesetzte tMCS-Systeme sind die venoarterielle extrakorporale Membranoxygenierung (VA-ECMO), die intraaortale Ballonpumpe (IABP) und die Impella-Herzpumpe (Impella CP® und Impella 5.5, Abiomed, Aachen, Deutschland) [1]. Bei der Impella handelt es sich um eine mikroaxiale Blutpumpe, die entweder perkutan über die Arteria femoralis (Impella CP) oder chirurgisch über die Arteria subclavia oder die Aorta ascendens eingebracht wird (Impella 5.5) und im linken Ventrikel zum Liegen kommt [4]. Die Impella saugt das Blut über einen Einlasskorb im linken Ventrikel an und wirft es über das Outlet-Areal direkt in die Aorta ascendens in physiologischer, antegrader Flussrichtung aus, was zu einer Entlastung des linken Ventrikels führt (Abb. 1; [5]). Im Gegensatz zur VA-ECMO kommt es daher bei der Impella zu einer Entlastung des linken Ventrikels, zu einer Senkung linksventrikulärer Füllungsdrücke, der mechanischen Herzarbeit und des myokardialen Sauerstoffverbrauchs. In der klinischen Praxis erreicht die Impella 5.5 ein HZV von bis zu 5,5 l/min, was bei den meisten Patienten dem vollen HZV entspricht. Derzeit ist die Impella 5.5 für eine Liegedauer von maximal 29 Tagen in der Europäischen Union zugelassen [5]. Die praktischen Erfahrungen haben allerdings gezeigt, dass auch eine längere Liegedauer (bis über 100 Tage) möglich ist [6]. Neben den temporären Systemen, kommen v. a. permanente invasive Herzunterstützungssysteme, wie das LVAD zum Einsatz.

Zu den wichtigsten Indikationen der Impella 5.5 zählen der kardiogene Schock, der Postkardiotomie kardiogene Schock (PCCS), das Low-Cardiac-Output-Syndrom (LCOS) und die akut dekompensierte Herzinsuffizienz sowie die Entlastung des linken Ventrikels an der VA-ECMO (ECMella) (Tab. 1; [5]). Die Entscheidungsfindung für den Einsatz der Herzpumpe wird auf Basis der erwarteten Dauer sowie der Flussrate zur notwendigen Herzunterstützung getroffen. Die Impella 5.5 kann als mittelfristige Überbrückung zur Evaluation und Vorbereitung weitergehender medizinischer Maßnahmen („bridge to decision“) wie eine anschließende Implantation eines dauerhaften Herzunterstützungssystems („bridge to LVAD“) oder eine Herztransplantation („bridge to transplant“ [HTx]) dienen oder bestenfalls das Erreichen einer vollständigen Erholung des nativen Herzens („bridge to recovery“) ermöglichen. Durch diese mittelfristige Unterstützung wird wertvolle Zeit gewonnen, in der sich das Herz erholen kann und das Behandlungsteam eine patientenindividuelle, optimale weitere Behandlungsstrategie entwickeln kann. Dabei ist keine intrathorakale Operation nötig, die ein deutlich invasiveres Vorgehen mit potenziell erneutem Myokardschaden bedeuten würde.

**Tab. 1:** Hauptindikationen von Impella 5.5 in Österreich

Indikationen für Impella 5.5
*Kardiogener Schock* SCAI Stadium C bis E Primäre Therapie oder als Eskalation von einem anderen kurzzeitigen tMCS (IABP, Impella CP oder VA-ECMO)
*Postkardiotomie kardiogener Schock*
*Low-Cardiac-Output-Syndrom*
*Akut dekompensierte Herzinsuffizienz*
*Fortgeschrittene Herzinsuffizienz* „Bridge to LVAD“ „Bridge to HTx“
*Linksventrikuläre Entlastung während der VA-ECMO Anwendung (ECMella)*

*tMCS* temporäres mechanisch zirkulatorisches Unterstützungssystem, *IABP* Intraaortale Ballonpumpe, *VA-ECMO* venoarterielle extrakorporale Membranoxygenierung, *LVAD* left ventricular assist device, *HTx* Herztransplantation

**Abb. 2:**
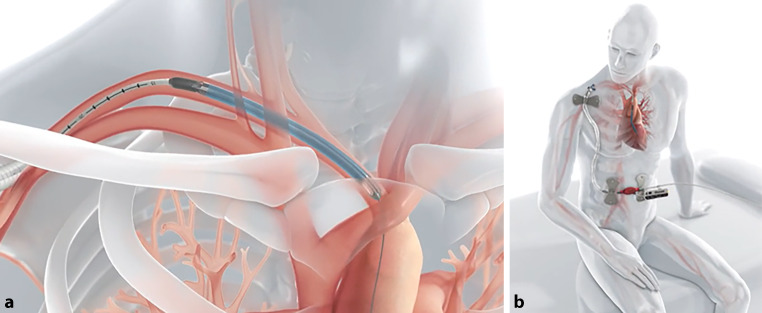
Einbringen der Impella 5.5. **a** Die Impella 5.5 wird mittels minimal-invasiver chirurgischer Technik über die Arteria subclavia eingesetzt. **b** Sie stellt ein mittelfristiges tMCS zur raschen Mobilisierung der Patienten dar. (Quelle der Abbildung: https://www.abiomed.de/ueber-uns/presse-und-news/media-kit)

Die Impella CP wird primär perkutan zur raschen Stabilisierung der Patienten im kardiogenen Schock eingesetzt. Eine Eskalation auf die Impella 5.5 sollte nach unserer Erfahrung bereits nach maximal 48 Stunden erfolgen, um eine ausreichende Flussrate zu erreichen, die Gefahr von Komplikationen zu minimieren und die Patientenmobilisierung zu ermöglichen (Abb. 2; [4]). Allerdings ist unter bestimmten Umständen die Einbringung einer Impella 5.5 auch als First-line-Therapie möglich. In unserer Praxis hat sich gezeigt, dass deutlich längere Liegezeiten, über die zugelassene Liegedauer von maximal 29 Tagen hinaus [5], möglich und sicher sind. Somit kann das Zeitfenster bis zu einer Entscheidung der notwendigen Therapie für die Patienten, wie etwa die dauerhafte Versorgung mit einem LVAD oder eine Herztransplantation, entsprechend ausgeweitet werden.

## Überblick über kürzlich veröffentlichte Studien

Eine rezente systematische Metaanalyse fokussiert auf unerwünschte Ereignisse und Überleben bei Patienten, die von September 2019 bis März 2023 mit Impella 5.0® (Abiomed, Aachen, Deutschland) oder Impella 5.5 therapiert wurden [7]. Studien, die für eine qualitative Auswertung ausgewählt wurden, zeigten eine heterogene Patientenpopulation mit der Mehrheit der Patienten mit akutem Myokardinfarkt mit kardiogenem Schock (AMICS) und PCCS. Die gepoolte Überlebensrate bis zur Entlassung aus dem Krankenhaus betrug 68 % (95 % Konfidenzintervall, CI, 58–78 %), und das 30-Tage-Überleben war bei 65 % (95 % CI, 56–74 %). Interessanterweise lag die Überlebensrate bis zur Entlassung aus dem Krankenhaus bei Patienten, die mit der Impella 5.5 behandelt wurden, bei 78 % (95 % CI, 72–82 %) [7]. Insgesamt stimmen diese Ergebnisse mit den Daten aus einer Analyse des National Inpatient Sample, welches eine repräsentative US-Datenbank ist, überein. In dieser Studie war die Sterblichkeitsrate im Krankenhaus bei rund 31 % bei Patienten, die eine Impella CP, 2.5® (Abiomed, Aachen, Deutschland), 5.0 und/oder 5.5 erhielten [8]. Eine ähnliche Analyse einer Qualitätssicherungsdatenbank der USA zeigte eine Überlebensrate von 80,5 % bei Patienten, die mit Impella 5.5 behandelt wurden [4]. Basierend auf diesen Daten und unserer Erfahrung, spekulieren wir, dass die Überlebensrate in dieser kritisch kranken Population signifikant höher wäre, wenn sie mit Impella 5.5 anstatt mit der zwischenzeitlich nicht mehr erhältlichen Impella 5.0 therapiert werden. Dies sollte auch der Fokus der zukünftigen Studien sein.

Die Danish German Cardiogenic Shock (DanGer Shock)-Studie (NCT 01633502) zeigte als erste randomisierte kontrollierte Studie einen Überlebensvorteil für ein tMCS mit kurzfristiger Unterstützungsdauer bei Patienten mit kardiogenem Schock nach ST-Hebungsinfarkt (STEMI) [9]. Die Studie verglich die Anwendung einer Impella CP im STEMI-induzierten kardiogenen Schock vs. der Standardtherapie ohne tMCS zusätzlich zur medikamentösen Therapie in beiden Studiengruppen. Während 15,6 % der Patienten in der Impella CP-Gruppe ein zusätzliches Device zur mechanischen Kreislaufunterstützung (überwiegend eine VA-ECMO) als Eskalation benötigten, erhielten 21,0 % in der Standardtherapiegruppe im Verlauf ein tMCS. Der primäre Endpunkt, die Gesamtmortalität nach 180 Tagen, konnte in der Impella CP-Gruppe gegenüber der Standardtherapiegruppe statistisch signifikant reduziert werden (45,8 % vs. 58,5 %, HR 0,74; 95 % CI 0,55–0,99; *p* = 0,04). Die absolute Reduktion des Sterberisikos für die Impella CP-Gruppe von 12,7 Prozentpunkten bedeutet eine „number needed to treat“ von 8. Beim kombinierten Sicherheitsendpunkt traten in der Impella CP-Gruppe deutlich mehr Komplikationen als in der Standardtherapiegruppe auf (24,0 % vs. 6,2 %, RR 4,74; 95 % CI 2,36–9,55). Diese höheren Raten an schweren Komplikationen, insbesondere moderate/schwere Blutungen (21,8 % vs. 11,9 %, RR 2,06; 95 % CI 1,15–3,66), Beinischämie (5,6 % vs. 1,1 %, RR 5,15; 95 % CI 1,11–23,84) und Nierenersatztherapie (41,9 % vs. 26,7 %, RR 1,98; 95 % CI 1,27–3,09) haben jedoch den Vorteil der tMCS-Therapie nicht überschattet [9].

## Guidelines und Empfehlungen

Trotz des Mangels an randomisierten Studien empfehlen internationale wissenschaftliche Gesellschaften in ihren Leitlinien und verschiedenen Konsenserklärungen im Allgemeinen die Verwendung der Impella 5.0/5.5 unter bestimmten Bedingungen für ausgewählte Patienten. Die Society of Cardiovascular Angiography and Interventions (SCAI) hat in einem Consensus Statement von 2019 fünf Schockstadien klassifiziert. Demnach sollen tMCS in den Schockstadien C (klassisch), D (verschlechternd) und E (extrem) innerhalb von 6 Stunden zum Einsatz kommen [10]. Die European Society of Cardiology (ESC) hat in ihren Leitlinien 2021 zur akuten und chronischen Herzinsuffizienz die tMCS-Therapie von einer Klasse-IIb-Empfehlung/Evidenzlevel C auf eine Klasse IIa/C ohne neue Studienevidenz angehoben [11]. Im Gegensatz dazu steht die IABP, die aufgrund der negativen IABP-SHOCK II-Studie nicht mehr zum Routineeinsatz empfohlen wird (Klasse III/B) [12].

Im gemeinsamen Konsensus-Statement von der European Association for Cardio-Thoracic Surgery (EACTS), der Extracorporeal Life Support Organization (ELSO), der Society of Thoracic Surgeons (STS) und der American Association for Thoracic Surgery (AATS) [13] besteht für die Impella 5.0, eine IIb/C-Empfehlung für Patienten als Ersttherapie oder als gemeinsame Behandlung mit einer VA-ECMO bei Vorliegen einer schweren isolierten linksventrikulären Dysfunktion. Die Guidelines der International Society for Heart and Lung Transplantation (ISHLT) und die Heart Failure Society of America on Acute Mechanical Circulatory Support empfehlen den Akuteinsatz von MCS im Fall eines kardiogenen Schocks SCAI Stadium C und D mit einer Klasse I/Level B [14]. Dennoch bleibt die Mortalität in dieser Patientenpopulation hoch, was darauf hindeutet, dass eine Verfeinerung und kohärentere Leitlinien erforderlich sind.

In Bezug auf Impella wurden Studien, die mit der Impella 5.0 durchgeführt wurden, in die Leitlinien aufgenommen. Aufgrund der Produktähnlichkeiten zur Impella 5.0 und der weiteren technischen Verbesserungen von Impella 5.5 gehen wir davon aus, dass die gleichen Leitlinien für die Verwendung von Impella 5.5 angewendet werden können, obwohl Daten erforderlich sind, um dies zu bestätigen. Darüber hinaus liegen zwar eine Reihe von einzel- und multizentrischen Studiendaten und verschiedene Übersichtsarbeiten zur Anwendung von tMCS mit Impella 5.5 vor [7], randomisierte klinische Studien in homogenen Patientenpopulationen mit Impella 5.5-Behandlung sind jedoch noch ausständig.

## Diskussion

Die mikroaxiale Flusspumpe Impella 5.5 erreicht mit der Entlastung des linken Ventrikels im Ausmaß des vollen HZV eine adäquate Kreislaufunterstützung für den akuten und chronischen Herzinsuffizienzpatienten [4]. Durch die Reduktion der linksventrikulären Pumparbeit des nativen Herzens wird die Chance auf Erholung erhalten, insbesondere im akuten Setting, und ermöglicht eine frühe Beurteilung des residuellen Zustands der Myokardfunktion. Da die Impella 5.5 über die Arteria subclavia oder über die Aorta ascendens eingebracht wird, werden Komplikationen signifikant reduziert, die bei anderen über den femoralen Zugang eingeführten tMCS-Systemen häufiger auftreten (z. B. Beinischämie) [15]. Darüber hinaus erlaubt diese Insertionstechnik sogar die Extubierung und Mobilisierung der Patienten. Zusätzlich verringert das Design der Impella 5.5 das Risiko für Hämolyse und sorgt für Lagestabilität [4].

Wenngleich die Daten aus der DanGer Shock-Studie [9] vielversprechend sind, stellen wir die Hypothese auf, dass die sofortige Einbringung einer Impella 5.5 oder eine raschere Eskalation von der Impella CP auf die Impella 5.5 das Ergebnis potenziell noch hätte verbessern können. Diese Spekulation beruht größtenteils auf der Tatsache, dass Patienten im schweren kardiogenen Schock typischerweise eine noch potentere Unterstützung benötigen, was die Impella 5.5 mit einem Spitzenfluss bis 5,5 l/min erreichen kann.

In Österreich kommt die Kombinationstherapie der ECMella auf 2 Arten zum Einsatz. Erstens kann Impella 5.5 als Unloading-Strategie vor oder nach der VA-ECMO-Implantation zur Nachlastverringerung eingesetzt werden [16]. Zweitens kann die VA-ECMO als Eskalationsstrategie bei vorher eingebrachter Impella 5.5 dienen, insbesondere wenn die Patientensituation hohe Flüsse und/oder Oxygenierung und/oder eine Rechtsherzunterstützung notwendig macht [17].

Wir glauben, dass die Möglichkeit der mittelfristigen Unterstützung mittels der Impella 5.5 eine therapeutische Lücke schließt, indem sie die Optimierung der Herzfunktion während des Wartens auf eine Herztransplantation zulässt, ohne das Risiko für Komplikationen zu erhöhen. Der Zeitgewinn durch den mittelfristigen Einsatz einer Impella 5.5 ermöglicht es daher, über weitere Behandlungsstrategien nachzudenken („bridge to decision“, „bridge to LVAD“, „bridge to HTx“), während die Option einer vollständigen Genesung des nativen Herzens („bridge to recovery“) aufrecht bleibt.

Der Einsatz der Impella 5.5 als protektives Postkardiotomie-tMCS („protected cardiac surgery“) ist in Europa ein aufstrebendes Therapiekonzept. Bei der tMCS-gestützten Mitralklappenreparatur wird das tMCS direkt im OP eingebracht, um den Patienten von der Herz-Lungen-Maschine zu entwöhnen. Die Impella 5.5 unterstützt den Patienten auf der Intensivstation bis zur Extubation und darüber hinaus, und könnte damit neue lebensrettende Möglichkeiten in Österreich eröffnen. Daten von der IMpella-Protected cArdiaC surgery Trial in Europe [18] (IMPACT EU, NCT05756751) und dem Register Impella in Cardiac Surgery [19] (ImCarS, DRKS00024560) werden mit Spannung erwartet, um der bereits identifizierten Evidenzlücke zu begegnen. IMPACT EU untersucht die Wirksamkeit und Sicherheit des perioperativen Impella 5.5-Einsatzes bei Hochrisikopatienten mit schwerer linksventrikulärer Dysfunktion, die am Herzen unter Herz-Lungen-Maschine operiert werden müssen. In diese prospektive, nicht randomisierte Studie sollen 123 Patienten eingeschlossen werden. Der primäre Endpunkt untersucht die Rate an Postkardiotomieversagen bis zur Krankenhausentlassung und sekundär die Gesamtmortalität und die Rate an Schlaganfällen [18]. Das ImCarS-Register untersucht die Qualität der chirurgischen Praxis und das klinische Management von Patienten, die mit tMCS-Geräten wie Impella 5.0/5.5 oder Impella RP® (Abiomed, Aachen, Deutschland; zur Rechtsherzunterstützung) mit oder ohne VA-ECMO unterstützt werden. Als Ergebnisse werden das Überleben bis zur Entlassung oder der nächsten Therapie und die Gesamtsterblichkeit nach einem Monat, mittelfristig nach 3 und 9 Monaten sowie nach einem Jahr analysiert [19].

## Conclusio

In der Praxis schließt Impella 5.5 die Lücke zwischen einer tMCS-Kurzzeitversorgung (z. B. mit Impella CP) und der Langzeitversorgung mit einem dauerhaft implantierten LVAD oder einem Spenderherz. Aufgrund der zugelassenen Liegedauer von bis zu 29 Tagen, die international und auch in Österreichs Zentren, weil häufig komplikationsarm, überschritten wird, stellt die Impella 5.5 eine mittelfristige Versorgungsmöglichkeit dar. Bei der Kombinationstherapie als ECMella erleichtert die Impella 5.5 die Entwöhnung von der VA-ECMO und kann die Extubation und frühere Mobilisierung der Patienten ermöglichen, was in der Folge eine kürzere Belagsdauer auf Intensivstation bewirken kann. Folglich ist die chirurgisch eingebrachte Impella 5.5 der Standard of Care zur mittelfristigen Therapie in Österreich von Patienten mit kardiogenem Schock und für Bridging-Szenarien wie „bridge to recovery“, „bridge to LVAD“, „bridge to decision“ und „bridge to HTx“.
